# A predictive model of Health Related Quality of life of parents of chronically ill children: the importance of care-dependency of their child and their support system

**DOI:** 10.1186/1477-7525-7-72

**Published:** 2009-07-28

**Authors:** Janneke Hatzmann, Heleen Maurice-Stam, Hugo SA Heymans, Martha A Grootenhuis

**Affiliations:** 1 Psycho Social Department, Emma Children's Hospital, Academic Medical Center (AMC), University of Amsterdam, Amsterdam, The Netherlands; 2 Department of Pediatrics, Emma Children's Hospital, AMC; University of Amsterdam, The Netherlands

## Abstract

**Background:**

Parents of chronically ill children are at risk for a lower Health Related Quality of Life (HRQoL). Insight in the dynamics of factors influencing parental HRQoL is necessary for development of interventions. Aim of the present study was to explore the influence of demographic and disease related factors on parental HRQoL, mediated by employment, income, leisure time, holiday and emotional support in a comprehensive model.

**Methods:**

In a cross-sectional design, 543 parents of chronically ill children completed questionnaires. A conceptual model of parental HRQoL was developed. Structural equation modeling was performed to explore the relations in the conceptual model, and to test if the model fitted the data.

**Results:**

The model fitted the data closely (CHISQ(14) = 11.37, p = 0.66; RMSEA = 0.0, 90%CI [0.00;0.034]. The effect of socio-demographic and medical data on HRQoL was mediated by days on holiday (MCS: β = .21) and emotional support (PCS: β = .14; MCS: β = .28). Also, female gender (β = -.10), age (β = .10), being chronically ill as a parent (β = -.34), and care dependency of the child (β = -.14; β = -.15) were directly related to parental HRQoL.

**Conclusion:**

The final model was slightly different from the conceptual model. Main factors explaining parental HRQoL seemed to be emotional support, care dependency, days on holiday and being chronically ill as a parent. Holiday and emotional support mediated the effect of demographic and disease-related factors on HRQoL. Hours of employment, leisure time and household income did not mediate between background characteristics and HRQoL, contrasting the hypotheses.

## Background

With the increased prevalence and incidence of chronic illness in children [1], the number of families with a chronically ill child has also increased. This increase is combined with a transfer of increasingly complex medical care to the home-situation (e.g. dialysis, parenteral nutrition). Also, family demographics have changed the last decades into smaller families, more single-parent families and mothers more often are employed [2]. These changes stress the need for a better understanding of the consequences for families caring for a chronically ill child. Caregiving demands can be extensive, and may lead to adverse psychosocial consequences for parents.

In a previous report we have shown that almost half of the parents of chronically ill children are at risk for an impaired Health Related Quality of Life (HRQoL) [3], particularly concerning vitality, sleep, daily activities, social functioning and depressive emotions. Other studies have also found similar results [4-6]. It is important to understand the dynamics of parental HRQoL, as parental mental functioning is known to influence their children's health and adjustment [7,8]. Furthermore, it contributes to the development of interventions to improve parental HRQoL. Up to now, most studies explored direct predictors of parental well-being and HRQoL, and positive associations were found with higher socio-economic status, coping style, few child behavior problems, less care giving demands, more social support, and an older age [4,9-11]. In addition to these direct associations, different conceptual frameworks have been developed in which demographic, medical and social variables not only directly but also indirectly influence health and well-being [10,12-14]. To our knowledge, most models address adaptation to illness in disease populations in children or adults, and only few models focus on parental well-being [11,14,15]. Raina et al, and King et al. developed and tested conceptual models of caregiver process and caregiver burden [10,11,14] including socioeconomic status, child characteristics, caregiver strain, psychosocial and coping factors.

**Figure 1:**
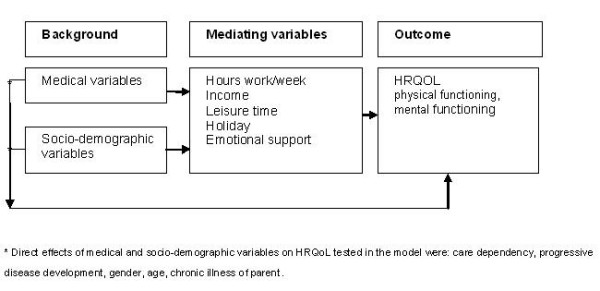
**Conceptual model of demographic, disease related and social factors influencing HRQOL**.

In line with these models, we suppose that HRQoL of parents of chronically ill children is influenced by demographic, disease related, and social factors, and that the influence these factors have is a dynamic process. With respect to social factors, parents of chronically ill children are known with lower employment rates and reduced leisure time activities compared to parents of healthy children [16,17]. Employment is related to parental well-being, Warfield [18] found that having a satisfying job reduces parental stress levels and Thyen et al [19] found worse mental health in unemployed mothers. The aim of the present study was to extend the literature by including these social variables in a comprehensive model explaining parental HRQoL. Therefore we constructed a model in which we described the influence of demographic and disease related variables on parental HRQoL, mediated by employment, leisure time, income, holiday and emotional support (Figure 1). We hypothesized that demographic and disease related characteristics (background) influenced employment, leisure time, income, holiday and emotional support (mediators). And that these mediators influenced caregiver mental and physical Health Related Quality of Life (HRQoL). We also hypothesized that age, gender and having a chronic illness themselves influenced HRQoL directly. The model of parental HRQoL was explored using structural equation modeling.

## Methods

### Participants and procedure

Parents of chronically ill children participated in this study, named the Care-project. Chronic illness in childhood was defined according to Mokkink et al. [20,21] using the following criteria: the disease occurs in children aged 0–18 years, the diagnosis is based on medical scientific knowledge, is not (yet) curable and exists for at least three months, or will probably endure longer, or at least three disease episodes have occurred the last year. According to the definition we selected ten different chronic diseases in childhood: asthma, diabetes, Down syndrome, Duchenne muscular dystrophy, end stage renal disease, metabolic diseases, profound multiple handicaps, sickle cell disease, spina bifida, and survivors of a brain tumor. Inclusion criteria were: [1] the chronically ill children were aged between 1–19 years, [2] were diagnosed >1 year before inclusion in the study, [3] the children lived at home, [4] parents were able to fill out the questionnaire in Dutch or English.

Between January 2006 and September 2007 parents of chronically ill children were invited to participate in the Care project in the Emma Children's Hospital/AMC in Amsterdam, The Netherlands, and through patient organizations. Parents received an introductory letter explaining the aim of the study and asking their participation. Parents decided themselves whether the mother or the father completed the questionnaire. The letter was accompanied by the questionnaire, an informed consent form and a stamped self-addressed envelope. Each family received one questionnaire, which was completed at home. The specific procedure for each disease group is described in Hatzmann et al [3]. The study was approved by the Medical Ethics Committee of the Academic Medical Center Amsterdam.

### Measurement

A self report questionnaire was developed for the Care project, including an existing HRQoL questionnaire, and questions regarding demographics, education, employment, child care, additional burden in the family (e.g. chronic illness of parents), use of health care services, leisure activities, and characteristics of the chronically ill child. Several questions were adapted from other studies [22-25]. The questionnaire was pre-tested with 15 parents of chronically ill children who met the inclusion criteria. Based on their suggestions, modifications were made to improve the survey's content and clarity. The questionnaire was also available in English, translated into English by a professional translator.

### Background variables: Demographic and disease related variables

Demographic variables included parental gender, educational level (low, intermediate, high), having a partner, country of birth (in The Netherlands, elsewhere), chronic illness in respondent themselves (y/n), chronic illness in their partner (y/n), age (parent and child), and number of children in the family. Disease related variables included parent report about disease development in their child in the previous year (progressive, improving, varying, stable), time since diagnosis (years), and dependency on daily care, defined as the number of life domains on which the child needs care (physical, mobility, eating & drinking, medication use, coping with devices, entertaining, contact with other children, education). This scale ranges from 0–8, where 0 indicates the child doesn't need support on the above mentioned domains, and score 8 indicates the child needs support on all domains.

### Mediating variables: work, income, leisure time, holiday and emotional support (social factors)

Mediating factors in our model were employment (hours per week), net household income (euro per month), hours per week spent doing leisure activities (sports, hobbies), holiday leave (number of days families went on holiday the last year), and emotional support (emotional support derived from partner, family, friends or neighbors, scored on 3-point scale: 0 = no support, 1 = more or less 2 = good support). The scale emotional support ranges from 0–8, where 0 indicates no support and 8 indicates good support.

### Outcome variable: HRQoL

HRQoL was assessed with the 'TNO-AZL Questionnaire for Adult's Health related Quality of life' (TAAQOL) [26]. The questionnaire measures health status problems weighted by the impact of problems on well-being on 12 multi-item scales: gross and fine motor functioning, cognitive functioning, sleep, pain, social functioning, daily activities, sexuality, vitality, positive emotions, depressive emotions and aggressiveness. Each item consists of two parts: the first part assesses the prevalence of a health problem or limitation in the past month, the second part the emotional response to the health problem or limitation. Answers were scored on 4 point scales. A single score is attributed to each combination of an item assessing the prevalence of a problem or limitation and the corresponding emotional response. The scales vitality, positive emotions, depressive emotions and aggressiveness only assess the occurrence of the feelings in the past month. Higher scores indicate a better HRQoL. The psychometric properties, validity and reliability, of the TAAQOL were satisfactory [26]. Overall physical and overall mental HRQoL were assessed by aggregation of all TAAQoL scale scores according to the algorithm described by Ware et al [27] which lead to the so-called Physical Component Score (PCS) and Mental Component Score (MCS). The relative contribution of each TAAQoL scale to MCS and PCS was derived from principal components analysis, non-orthogonal rotation (Oblimin), based on the assumption that physical and mental HRQoL are interdependent.

### Statistical analysis

First, HRQoL scales were constructed and missing data were imputed based on the guidelines of the TAAQOL. In calculation of the scale scores one missing combined-item score was allowed for, the missing score is replaced by the mean value of the non-missing item scores. In addition, missing HRQoL outcomes were handled through the Expectation-Maximization estimation method (SPSS 16.0).

Structural Equation Modeling (SEM), using LISREL 8.30, was performed to investigate the relationships among the variables in the conceptual model and to test whether the conceptual model fitted the data, using the correlation matrix. Standard SEM requirements of data to be continuous en multivariate normal distributed were checked as follows. First the distributions of the variables were inspected. Variables at the first level (demographic and medical variables) were dichotomised if necessary, e.g. educational level and disease development. The variables at the second level were inspected carefully and if necessary outliers were recoded, e.g. very high income scores were recoded to the highest income that was acceptable considering a normal distribution. The distribution of the dependent variables at the third level (HRQoL outcomes) appeared to be acceptable. After that, several regression analyses were performed (level 2 predicted by level 1, level 3 predicted by level 1 and 2) to check assumptions. We did not find any serious violation.

In SEM the covariance structure that follows from the proposed model is fitted to the observed covariances [28]. The maximum likelihood estimate method yields estimates of the regression coefficients in the model, standard errors, and a χ^2^-test of overall goodness-of-fit [29]. An alternative fit measure is the root mean square error of approximation (RMSEA). According to a generally accepted rule of thumb [30], RMSEA values lower than 0.08 indicate satisfactory fit, and values lower than 0.05 indicate close fit. In addition to overall goodness-of-fit, component fit was evaluated by inspecting standardized discrepancies between observed and expected correlations, and LISREL's modification indices [29]. We used a significance level of p < 0.05 for the regression coefficients. Standardized regression coefficients of 0.1 were considered small, 0.3 medium and 0.5 large [31]. For binary coded predictor variables, regression coefficients of 0.2 can be considered small, 0.5 medium and 0.8 large.

## Results

### Participants

A total of 1106 parents of chronically ill children from ten different diagnosis groups were asked to participate in the Care project, of which 580 (52%) completed the Care-questionnaire. The response for each diagnosis group is described in Hatzmann et al [3]. After estimation of missing data, the full HRQoL data of 543 (49%) parents was available for analysis. Non-responders did not differ from responders (p < 0.1) with respect to the age and gender of the children, except for the children with asthma and sickle cell disease, with both more boys in the non-responders group (p < 0.1).

The mean age of the caregivers was 42 (SD: 6.5) years, of which 83% was female (Table 1). Most respondents had a partner (86%) and were born in the Netherlands (82%). Fourteen percent of the respondents had a chronic illness themselves, and 10% of their partners. Families had on average 2.3 (SD: 0.9) children. The chronically ill children were on average 10.0 (SD 4.4) years, and mean time since diagnosis was 7.9 (SD 4.2) years (Table 2).

**Table 1:** Characteristics of observed variables of caregivers and their chronically ill children

	Parents chronically ill children	Sample size
**Demographic Variables**	n (%)	

Gender (Female)	452 (83)	542
Educational level^1^		535
Lower	140 (26)	
Intermediate	220 (41)	
Higher	175 (33)	
Married/Partner	469 (87)	541
Born in the Netherlands	445 (82)	543
Respondent chronically ill	77 (14)	542
Partner Chronically ill	52 (10)	542
	Mean (sd)	
Age Parent Years (SD)	42.0 (6.5)	540
Children per family	2.3 (0.9)	533
Age child	10.0 (4.4)	532
**Disease related variables**		
Care Dependency *	3.2 (2.5)	540
Time since diagnosis	7.9 (4.2)	496
Disease Development	n (%)	511
Progressive	93 (18)	
Improving	134 (26)	
Varying	120 (23)	
Stable	164 (32)	

**Social participation**	Mean (SD)	

Hours of work/week	15.38 (13.89)	497
Monthly family income (euro)	2504 (1171)	480
Leisure time (hours)	4.9 (5.7)	539
Holiday (days/year)	19.3 (11.6)	476
Emotional support **	4.8 (2.0)	529

^1 ^Highest level completed. Lower: Primary education, Lower and Middle General Secondary education; Intermediate: Middle Vocational education, Higher Secondary education, Pre-university education; Higher: Higher Vocational Education, University

* scale 0–8 (high score representing high dependency)

** scale 0–8 (high score representing good support)

**Table 2:** Characteristics of the chronically ill children

Age (mean, sd)	10.0 (4.4)
Time since diagnosis in years (mean, sd)	7.9 (4.2)
	
Diagnosis:	n (%)
Asthma	90 (17)
Survivors of brain tumor	38 (7)
Diabetes	24 (4)
Down syndrome	101 (19)
Duchenne muscular dystrophy	57 (10)
End stage renal disease	21 (5)
Metabolic diseases	118 (22)
Profound complex handicap	13 (2)
Sickle cell disease	61 (11)
Spina Bifida	20 (4)

### Model fit

**Figure 2:**
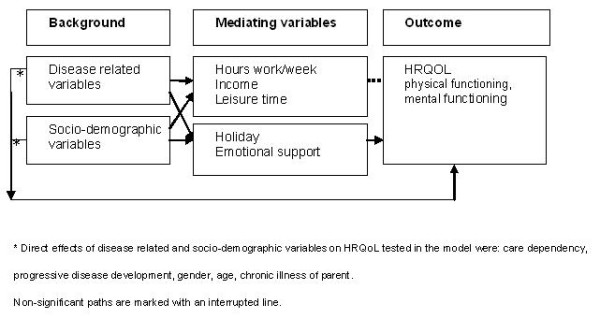
**Modified final model of predicting parental HRQOL of caregivers of chronically ill children**.

The conceptual model (Figure 1) was fitted to the correlation matrix. The CHISQ measure of overall goodness-of-fit was 36.92 (CHISQ(18), p = 0.0054) and the hypotheses of exact fit was rejected. The RMSEA was 0.044, and the 90% confidence interval (CI) ranged from 0.023 to 0.064, which indicated that the fit was satisfactory. Inspection of component fit indices indicated two possible modifications. The modification indices suggested an additional direct effect of "care dependency" and "worsening disease development" on HRQoL. These modifications were added to the model, resulting in a modified model with close fit: CHISQ(14) = 8.70, p = 0.085; RMSEA = 0.0, 90% CI [0.00;0.023]; CFI = 1.00. The modified model explained 21% of the variance in PCS and 20% of the variance in MCS. Figure 2 gives a graphical display of the modified model, and Additional file 1; Table S1 gives the parameter estimates.

The first part (1) of Additional file 1; Table S1 presents the effects of demographic and disease related variables (background characteristics) on the mediating factors, the second part (2) contains the direct effects of the background characteristics on HRQoL, and the third part (3) contains the effects of the mediating factors on HRQoL. The total effect of a variable on HRQoL can be calculated using the direct and indirect pathways in the modified model, as the following example illustrates. Additional file 1; Table S1 shows the direct and indirect effects of "care dependency" on MCS. First, an increase of one standard deviation on "care dependency" is associated with a statistically significant decrease of 0.15 standard deviation in MCS (direct effect). Second, an increase of one standard deviation on "care dependency" is associated with a statistically significant decrease of 0.13 standard deviation in "holiday", while the effect of "holiday" on MCS was 0.21. So the statistically significant effects of "care dependency" on MCS can be calculated as follows: -0.15 + (-0.13 × 0.21) = -0.18. Apart from that, the other direct and indirect effects of "care dependency" on MCS were small and non-significant, so that the total effect (-0.20) remains small.

### Effects of the demographic and disease characteristics

All the significant regression coefficients of the demographic and disease related variables were small to medium [31], ranging from β = 0.08 to β = 0.38. The demographic variables affected the mediating factors more strongly than the disease related variables, specifically: gender, educational level, country of birth, and living with a partner appeared to correlate with employment, leisure time, income, holiday and emotional support.

Several direct and indirect effects of demographic and disease related variables on HRQoL were found. Being female had a negative (direct) as well as positive (indirect, via "emotional support") effect on PCS. High educational level, living with a partner and being born in the Netherlands were correlated with better HRQoL, indirectly via "emotional support" (PCS and MCS) and "holiday" (MCS). Having a partner with a chronic disease is also indirectly associated with better MCS via "holiday".

On the contrary, suffering from a chronic disease by the parent and greater care dependency had a negative impact on HRQoL. More specific, parents who suffered from a chronic disease themselves or who reported greater care dependency, experienced worse PCS and MCS, directly and/or indirectly, via "holiday" and "emotional support".

### Effects of the mediating factors (work, income, leisure time, holiday and emotional support)

A few significant effects of mediating factors on HRQoL were found. The effects were rather small, ranging from β = 0.14 to β = 0.28. First, parents who experienced more emotional support reported better PCS as well as better MCS. Second, parents who went more days on holiday reported higher levels of MCS.

## Discussion

In the present study, a model explaining direct and indirect associations of demographic, disease related and social factors with the HRQoL of parents of chronically ill children was tested. In our model, parental HRQoL is directly associated with gender, parental age, having a chronic illness as a parent, care-dependency of the child, emotional support, and number of day's parents went on holiday in the past year. Socio-demographic variables mainly relate to HRQoL indirectly through holiday and emotional support. When looked at the size of the (significant) effects, dependency of the child, chronic illness of the parent, days on holiday, and the support system seem to be the main factors predicting parental HRQoL.

The final model fitted the data closely, but appeared to be slightly different from our conceptual model. As we hypothesized, demographic variables did influence work per week, family income and leisure time, and disease related variables influenced leisure time and holiday. But work, income and leisure time did not significantly mediate between demographic & disease related variables and HRQoL in the final model. Although several studies showed that parents of children with a chronic disease work fewer hours and spend fewer hours doing leisure activities [17,19,32], we have not found evidence that this influences HRQoL. A potential reason for the lack of effect is that not the amount, but satisfaction with work and leisure time is more important in explaining HRQoL [18].

Within the disease related variables, care-dependency was most associated with HRQoL. It influenced HRQoL both directly and indirectly through days on holiday per year. An increase in dependency of children on others for care, leads to a lower HRQoL in their parents. The importance of care dependency was also seen in other studies [11,17]. Unfortunately we do not know whether it is the emotional aspect of having a dependent child, or whether this is caused by the extra caregiving demands. A potential way to diminish the extra caregiving demands is making use of respite care. Drawn up from the literature, the effect of respite care on well-being of parents of chronically ill children is however not easily evaluated [33-36]. In caregivers in general (not only parents of ill children), some evidence regarding the effectiveness of respite care is found, and these caregivers expected that respite care would increase their well-being [35]. This implies that a good family support system or official support in terms of respite care would be able to improve caregiver HRQoL. However, more evidence within the population of parents of chronically ill children is needed.

In addition to the above described positive effect of practical support, emotional support has a positive influence on parental HRQoL. Emotional support was measured as an evaluation of the quality of support (no support, moderate or good emotional support) from partner, family, friends and neighbours. Other research also shows the importance of a support network for parental emotional well-being [37,38]. Parents getting the best emotional support were mothers with high education and a partner, born in the Netherlands. Hence, parents at risk for less support, and thus a lower HRQoL are single parents with lower education, not born in the Netherlands, and parents who are chronically ill themselves. In terms of prevention, parents should be stimulated to maintain and invest in their social network.

The number of days per year families went on holiday predicted the mental aspect of HRQoL positively. The present study does not distinguish between parents going on a holiday alone or with their children. Parents who went on holiday more often had a higher educational level, a partner and were born in The Netherlands. On the contrary, parents who were chronically ill themselves and parents whose children were more dependent on care, went less days on holiday per year. This group of parents already has a lower HRQoL, in addition, they also are less able to benefit from going on a holiday. More research is needed regarding if and how these parents can benefit from going on a holiday despite the disease related limitations.

The conceptual model used in this study only included mediating effects, while moderation was not tested. In our model, especially emotional support would theoretically be a plausible moderator of the effect of background characteristics on HRQoL. Despite plausibility, moderating effects of emotional support were not found. This is in line with a study by Quittner et al [39], who found evidence for a mediating effect and not for a moderating effect of social support on chronic parenting stress.

These results should be considered in light of a number of limitations. First, the response rate was 52%, with a majority of relatively good educated, Dutch, two-parent families. Therefore, our results are not representative for the entire group of parents of chronically ill children. Future research in other socio-demographic subgroups is necessary in order to provide adequate data and interventions for all parents. Second, although our model fitted the data closely, it explained only 21% of the variance of mental HRQoL and 20% of physical HRQoL, which might partly be due to the fact that we did not include factors as coping and family function. Other studies show that coping is an important variable in adaptation to chronic illness [13,40,41], and family function and child behavior also are associated with caregiver function [11,14]. Our model on the other hand shows the significance of care dependency and emotional support on parental HRQoL. Third, methodological limitations are the use of parent report and a single informant, which may lead to overestimation of the effects due to shared method variance. Also, the cross-sectional nature of the study does not allow inferences about causality. Our model should therefore be considered an explorative model, describing directions of associations, but not confirming causality. Fourth, using summary scores of HRQoL as a dependent variable had the advantage of its density and distribution. A disadvantage is the loss of detail in the analyses of HRQoL. The parents in the present study reported several problems on social and emotional domains[3], that are now summarized in mental and physical components of HRQoL. It is therefore important to realize where the summary scales are based on. Notwithstanding these limitations, the results of the present study give more insight in the dynamics of parental HRQoL.

## Conclusion

The final model fitted the data closely. Socio-demographic characteristics mainly influenced HRQoL indirectly by holiday and emotional support. Care-dependency and chronic illness of the parent had both direct and indirect negative effects on HRQoL. The significant effects were all small, meaning that we should be careful drawing conclusions based on these data.

### Implications for future research and clinical practice

Based on the results recommendations for future research include development of a more specific model with fewer variables and addition of psychosocial factors. Both mediating and moderating effects of these psychosocial variables should be considered. Also, future research should address quality of work and leisure time in addition to measures of quantity.

In clinical practice, insight in the factors that affect HRQoL may help health care providers to be aware of parents vulnerable for problems. The child and its family should be the focus of the professional. Also, professionals should be made aware of the consequences of care for parents during their training. Furthermore, they should be trained in how to detect and refer (e.g. to social worker of psychologist) parents who need professional support. Early detection and referral of parents at risk for impaired HRQoL could be achieved by using PRO's (Patient/Parent Reported Outcomes) in both outpatient and clinical settings [42]. For now, professionals should be aware of parents with lower socio-economic status, who are chronically ill themselves and have children with higher levels of care-dependency. Interventions should be directed at empowering parents to set up an adequate support system in order to derive emotional support and share the care for their children.

## Competing interests

The authors declare that they have no competing interests.

## Authors' contributions

JH designed the study, collected data, analyzed and interpreted the data and drafted the manuscript. HM analyzed and interpreted data, drafted and revised the manuscript. HH supervised design and execution of the study and revised the manuscript. MG designed and supervised execution of the study, analyzed and interpreted data and revised the manuscript. All authors read and approved the manuscript.

## Supplementary Material

Additional file 1 **Predictive model of Health-related Quality of life in parents of chronically ill children: standardized Regression Coefficients and Percentage of Explained Variance of the Modified Model**. Table showing the Predictive model of Health-related Quality of life in parents of chronically ill children: standardized Regression Coefficients and Percentage of explained variance of the modified model. The model explains 21% and 20% of the variance of PCS and MCS, respectively. Click here for file
