# Supplementary material for: A predictive model of Health Related Quality of life of parents of chronically ill children: the importance of care-dependency of their child and their support system
Source: Health Qual Life Outcomes. 2009 Jul 28;7:72. doi: 10.1186/1477-7525-7-72 (PMC2731060; doi:10.1186/1477-7525-7-72)
Supplement: Additional file 1 — Predictive model of Health-related Quality of life in parents of chronically ill children: standardized Regression Coefficients and Percentage of Explained Variance of the Modified Model. Table showing the Predictive model of Health-related Quality of life in parents of chronically ill children: standardized Regression Coefficients and Percentage of explained variance of the modified model. The model explains 21% and 20% of the variance of PCS and MCS, respectively. [file 1477-7525-7-72-S1.doc]

**Table S1 - Predictive model of Health-related Quality of life in parents of chronically ill children: standardized Regression Coefficients and Percentage of Explained Variance of the Modified Model***

|  |  | Gender:  Female | | Age parent | Educational level:  High | Partner | Country of Birth-NL | Chronic Illness self | Chronic Illness Partner | Number of children | Age ill child | Time since diagnosis | Care dependency | disease development-progressive | |
| --- | --- | --- | --- | --- | --- | --- | --- | --- | --- | --- | --- | --- | --- | --- | --- |
| **1** | **Effects of demographic and disease related variables on the mediating factors** | | | | | | | | | | | | | | |
|  | Hours of work/week | | **-.38** | .00 | **.17** | .05 | .04 | -.05 | .05 | **-.08** | .07 | -.02 | -.07 | -.04 | |
|  | Monthly family income | | -.02 | **.10** | **.33** | **.22** | **.16** | .00 | -.06 | -.02 | -.05 | .00 | -.04 | .05 | |
|  | Hours leisure time/week | | **-.08** | .07 | .05 | .07 | **.15** | .08 | .02 | -.03 | .00 | **.14** | -.02 | **-.10** | |
|  | Holiday (days)/year | | -.07 | .06 | **.22** | **.19** | **.11** | **-.10** | **.08** | -.04 | -.03 | .06 | **-.13** | .00 | |
|  | Emotional support | | **.11** | **-.09** | **.10** | **.33** | **. 28** | **-.12** | -.03 | -.01 | -.07 | .08 | -.06 | -.03 | |
| **2** | **Effects of demographic and disease related variables on parental HRQoL** | | | | | | | | | | | | | | |
|  | Physical functioning (PCS) | **-.10** | | .03 |  |  |  | **-.34** |  |  |  |  | **-.14** | -.02 |  |
|  | Mental functioning (MCS) | -.04 | | **.10** |  |  |  | -.05 |  |  |  |  | **-.15** | -.08 |  |
|  |  | Hours work | | Family Income | Leisure time/  week | Holiday | Emotional support |  |  |  |  |  |  |  | *Explained*  *Variance* **§** |
| **3** | **Effects of mediating factors on HRQoL** | | | | | | | | |  |  |  |  |  |  |
|  | Physical functioning (PCS) | .06 | | .08 | .05 | .03 | **.14** |  |  |  |  |  |  |  | *0.21* |
|  | Mental functioning (MCS) | -.01 | | -.06 | .07 | **.21** | **.28** |  |  |  |  |  |  |  | *0.20* |

* N = 543; overall goodness of fit CHISQ(14) = 8.70, p = 0.85; RMSEA = 0.0, 90% confidence interval [0.0;0.023]; CFI= 1.00. Bold regression coefficients differ significantly from zero at α = 0.05.

**§** The model explains 21% and 20% of the variance of PCS and MCS, respectively.

Empty boxes indicate that no effect was tested
